# New Insights into Sensitization Mechanism of the Doped Ce (IV) into Strontium Titanate

**DOI:** 10.3390/ma11040646

**Published:** 2018-04-23

**Authors:** Taiping Xie, Yuan Wang, Chenglun Liu, Longjun Xu

**Affiliations:** 1 Chongqing Key Laboratory of Extraordinary Bond Engineering and Advanced Materials Technology (EBEAM), Yangtze Normal University, Chongqing 408100, China; 2 State Key Laboratory of Coal Mine Disaster Dynamics and Control, Chongqing University, Chongqing 400044, China; xlclj@cqu.edu.cn; 3 Environmental Monitoring Center Station of Suining City, Suining 629000, China; wangyuan@163.com; 4 College of Chemistry and Chemical Engineering, Chongqing University, Chongqing 400044, China

**Keywords:** strontium titanate, Ce(IV), doping modification, photocatalyst

## Abstract

SrTiO_3_ and Ce^4+^ doped SrTiO_3_ were synthesized by a modified sol–gel process. The optimization synthesis parameters were obtained by a series of single factor experiments. Interesting phenomena are observable in Ce^4+^ doped SrTiO_3_ systems. Sr^2+^ in SrTiO_3_ system was replaced by Ce^4+^, which reduced the surface segregation of Ti^4+^, ameliorated agglomeration, increased specific surface area more than four times compared with pure SrTiO_3_, and enhanced quantum efficiency for SrTiO_3_. Results showed that Ce^4+^ doping increased the physical adsorption of H_2_O and adsorbed oxygen on the surface of SrTiO_3_, which produced additional catalytic active centers. Electrons on the 4f energy level for Ce^4+^ produced new energy states in the band gap of SrTiO_3_, which not only realized the use of visible light but also led to an easier separation between the photogenerated electrons and holes. Ce^4+^ repeatedly captured photoelectrons to produce Ce^3+^, which inhibited the recombination between photogenerated electrons and holes as well as prolonged their lifetime; it also enhanced quantum efficiency for SrTiO_3_. The methylene blue (MB) degradation efficiency reached 98.7% using 3 mol % Ce^4+^ doped SrTiO_3_ as a photocatalyst, indicating highly photocatalytic activity.

## 1. Introduction

SrTiO_3_ is a good stability photocatalyst, possessing a relative narrow band gap. It is a promising function material which has been widely used in water decomposition to produce hydrogen, degradation of organic pollutants, and photochemical cells fields [1].

The excitation wavelength for SrTiO_3_ is 387 nm, which places it in UV light range. Visible light range is from 400 to 700 nm, occupying 50% of the solar light spectrum [2]. Therefore, an extension of the absorption light range of SrTiO_3_ into visible light may promote additional practical applications.

Many researchers have successfully prepared nano-structured SrTiO_3_ by various synthesis methods. However, these methods have several disadvantages; for example, low yield, high pollution, high cost, and complicated preparation processes. Accordingly, an exploration of environmentally friendly and low cost synthesis approaches for nano-SrTiO_3_ is important.

The function of doped ions is to capture and release photogenerated carriers quickly [3], thereby controlling the diffusion of photogenerated carriers in the catalyst particles, increasing the lifetime of photogenerated carriers, and improving the photocatalytic performance of a single-component photocatalyst.

The unique electronic structure of rare earth elements provides several thermodynamic and kinetic advantages [4]. Such elements have variable valence oxidation states under certain conditions. In the photocatalytic reaction process, variable valence can effectively transfer photogenerated electrons to prolong the time needed for the effective separation between photogenerated electrons and holes. Concurrently, the amount of rare earth elements can change the band gap of a single-component photocatalyst and expand its light absorption range.

The rare earth element cerium (Ce) can produce multiple electronic configurations and absorb visible light. It is the ideal application by which to dope elements for photocatalyst modification. Previous research has suggested [5,6,7,8,9] electron transfer between Ce^4+^ and Ce^3+^ may enhance photocatalytic activity of ZnO, Al_2_O_3_, and TiO_2_ as a result of the Ce dopant capturing the photogenerated electrons and suppressing the recombination of electron–hole pairs. In addition, an appropriate amount of doped Ce may enhance the property of corrosion resistance to ensure reuse for the modified products. Nevertheless, modification research for SrTiO_3_ by doping Ce^4+^ has not yet been reported.

Here, we report an environmentally friendly and low cost synthesis method with a simple preparation process in which Ce^4+^ doped SrTiO_3_ in situ synthesis was carried out to modify SrTiO_3_.

## 2. Experimental Procedures

All reagents were of analytical grade purity and used directly without further purification except that of SrCO_3_. The water used in all experiment processes was deionized water.

### 2.1. Preparation of Pure SrTiO_3_

SrCO_3_ was prepared according to the sol–gel method. A transparent mixed solution (marked A), including 1.47 g SrCO_3_ and tartaric acid with different ratios, was prepared by mixing and stirring at 60 °C for 10 min. Subsequently, 3.4 mL butyl titanate was added to the (A) solution under stirring conditions at 80 °C to form a yellow homogeneous sol. The prepared homogeneous sol was dried at 90 °C for 10 h to obtain dry gel. The resultant product was obtained by calcining the dry gel at 800 °C for 2 h.

### 2.2. Preparation of Ce-Doped SrTiO_3_

Ce-doped SrTiO_3_ was prepared according to the in situ sol–gel method. Under the optimum conditions of SrTiO_3_ preparation, ammonium ceric nitrate was added to the (A) solution. The addition amount (x) was 1 mol %~4 mol %. Other processes were not changed. It could be observed from the resultant product that an increase in the amount of ammonium ceric nitrate doped corresponded to a gradually deepening orange color in the product.

### 2.3. Materials Characterizations

Fourier transform infrared spectroscopy (FTIR) spectra of samples were recorded on an FTIR (Perkin-Elmersystem 2000, Akron, OH, USA) spectrometer using KBr powder-pressed pellets. Phase identification via X-ray diffraction (XRD) was conducted on an X-ray diffractometer (Shimadzu, XRD-6000, Kyoto, Japan) using Cu K_α_ irradiation at a scanning rate of 4°·min^−1^ with the 2θ range of 20°–70°. X-ray photoelectron spectroscopy (XPS) measurements were carried out on an XPS-XSAM800 (Kratos, Manchester, UK) spectrometer with an achromatic Al K_α_ X-ray source and an analytical chamber with a base pressure of 2 × 10^−7^ Pa. The X-ray gun was operated at 180W (12 kV, 15 mA). The Brunauer-Emmett-Teller (BET) special surface area was determined through N_2_ adsorption at 77 K using an adsorption instrument (ASAP-2020, Micromeritics, Norcross, GA, USA). The samples’ morphologies and microstructures were observed via a scanning electron microscopy (SEM, FEI, F50, ZEISS, Oberkochen, Germany) The UV-vis diffuse reflectance spectra (DRS) of samples were measured using a UV-vis spectrophotometer (TU1901, Beijing Purkinje, Beijing, China). BaSO_4_ was used as a reflectance standard.

### 2.4. Photocatalytic Activity Tests

100 mL of methylene blue (MB) aqueous solution with a given concentration and its corresponding photocatalyst dosage of 1 g/L were added into a quartz container and stirred for 1 h in the dark to reach the adsorption-desorption equilibrium. A 500 W Xe lamp equipped with UV cut-off filter was used as the visible light source (λ ≥ 420 nm). At the given irradiation time intervals, a series of the reaction solution was sampled and the absorption was measured with the UV-vis spectrophotometer (TU1901, Beijing Purkinje, Beijing, China).

#### 2.4.1. Effect of Initial Concentration of Methylene Blue on Photocatalytic Activity

According to the literature, initial concentration had a significant influence on the photocatalytic activity. A greater concentration of methylene blue produced a deeper color, which significantly affected the absorption of light irradiation for photocatalysts. Too low of a methylene blue concentration cannot fully reflect the photocatalytic activity. Therefore, to completely reveal photocatalytic activity of SrTiO_3_, a better initial concentration should be chosen via single factor experiment.

Here, we prepared a series of MB solutions with different concentrations (20 mg/L, 50 mg/L, 100 mg/L, 150 mg/L, and 200 mg/L). The degradation rate after 2 h reaction was tested using pure SrTiO_3_ and Ce-doped SrTiO_3_ as photocatalyst.

#### 2.4.2. Effect of Ce Doping Amount on Photocatalytic Activity

In order to investigate the effect of the Ce doping amount on the photocatalytic activity of SrTiO_3_, the photocatalysis mechanism was discussed and comparative experiments were performed. Four varieties of SrTiO_3_ with Ce content of 1 mol %–4 mol % were measured using photocatalytic reaction.

## 3. Results and Discussion

### 3.1. Optimization of Synthesis Conditions

#### 3.1.1. Effect of Tartaric Acid Dosage

In this synthesis process, tartaric acid was used as the solvent of SrCO_3_ and also as the complexing agent of tetrabutyl titanate. The following reaction occurred as a solvent for SrCO_3_:

CO_3_^2−^ + HOOCCHOHCHOHCOOH→CO_2_↑ + H_2_O + ^−^OOCCHOHCHOHCOO (1)

The formation of homogeneous sol–gel via the complexation reaction between butyl titanate and tartaric acid was divided into two steps:

First, the stable organic complex of Ti^4+^ was formed by mixing butyl titanate with tartaric acid under stirring condition.

Ti(OC_4_H_9_)_4_+xHOOCCHOHCHOHCOOH→Ti(OC_4_H_9_)_4-2x_+xHOCH_2_CHOHCHOHCH_2_OH (2)

Ti(OC_4_H_9_)_4_ + yHOOCCHOHCHOHCOOH + xH_2_O→Ti(OH)_x_(OOCCHOHCHOHCOO)_y_ + 4HOC_4_H_9_ (3)

Second, Ti^4+^ complexes could create a network structure in the sol–gel system by cross-linking action. In this process, Sr^2+^ ions were uniformly distributed in the sol–gel system by electrostatic action.

It can be seen from the reaction process that the amount of tartaric acid could not only dissolve SrCO_3_ but also ensure the complete complexation of Ti^4+^ with tartaric acid in order to obtain a stable organic complex including Ti^4+^.

When the amount of organic complexing agent was too small, the reaction system produced precipitation phenomenon; this resulted from the minimal presence of the complexing agent, which did not fully react with the metal ion. Accordingly, metal salt precipitation and crystallization made the formation of a clear and transparent sol more difficult.

In addition, tartaric acid also acts as a stabilizing agent in the formation of network-structured sol–gels through the cross-linking reaction between tartaric acid and Ti^4+^. In the sol–gel method process, the entire reaction system has no active group for polycondensation reaction.

The reaction between Ti^4+^ complex molecules was achieved only through the formation of hydrogen bonds from water molecules instead of polycondensation reaction. However, the hydrogen bond was not stable and easily disconnected. Accordingly, this kind of gel with cross-linking of hydrogen bonds easily absorbed water and subsequently was moisturized in the atmosphere. When the deliquescence gel was sintered at a high temperature, an undesired agglomeration phenomenon was observed in the resultant product. Fortunately, the tartaric acid contained numerous hydroxyl groups which could replace the hydrogen bonds via the crosslinking reaction process. Thus, a more stable, uniform transparent gel was formed.

Therefore, in the preparation process, the amount of tartaric acid must meet the needs of the solvent for SrCO_3_, the complexing agent of butyl titanate, and the stabilizer of the entire reaction system. However, an excessive amount of complexing agent in the preparation process might incur high costs.

To adjust the mole ratio of Sr^2+^ and tartaric acid to 2:1–1:5 in the preparation process of SrTiO_3_, the optimal dosage of tartaric acid was chosen by observing sol transparency and powder agglomeration after sintering, calculating relative crystallinity (The relative crystallinity can be obtained by processing XRD data of the sample.), and photocatalytic activity of the products. Table 1 lists the experimental phenomena and relative crystallinity of SrTiO_3_ in the system with different molar ratios of Sr^2+^ and tartaric acid (2:1–1:5).

As seen in Table 1, the molar ratio of Sr^2+^ and tartaric acid was higher than 1:1, the white precipitation was observed, and the relative crystallinity of the product was low. These phenomena indicated that tartaric acid added in the system was not sufficient to completely generate complexation reaction with Ti^4+^.

**Figure 1:**
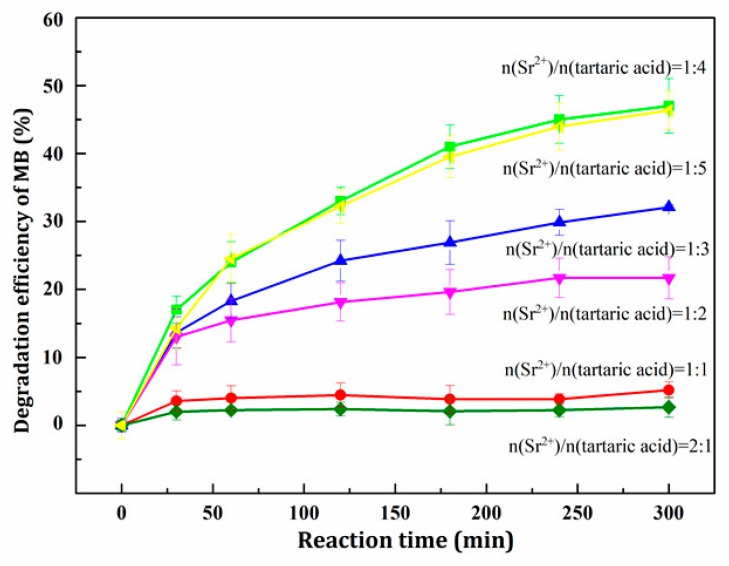
Effect of different dosage of tartaric acid on degradation efficiency of MB.

To further investigate the influence of tartaric acid dosage on the photocatalytic activity of SrTiO_3_, the samples were used in a photocatalytic activity test, and the experimental results are shown in Figure 1. It can be seen that the photocatalytic activity of SrTiO_3_ for methylene blue was greatly affected by the amount of tartaric acid. When the molar ratio of Sr^2+^ and tartaric acid was less than 1:4, the photocatalytic activity was highest. In contrast, when the molar ratio of Sr^2+^ and tartaric acid was 1:5, the photocatalytic activity was lower. Therefore, the optimum molar ratio of Sr^2+^ and tartaric acid was 1:4.

Concurrently, Table 1 and Figure 1 also demonstrate that the degradation rate of methylene blue was enhanced with the increase in the relative crystallinity of the as-prepared SrTiO_3_. This provided a theoretical basis for the modification of SrTiO_3_.

#### 3.1.2. Effect of Distilled Water Amount

The dissolution of SrCO_3_ and tartaric acid, the multistage ionization of tartaric acid, and the formation of sol–gel by the strontium titanate precursor all occurred in aqueous solution. Therefore, the amount of distilled water affected the preparation process of SrTiO_3_. When the water content in the solution was insufficient, the solution generated precipitation phenomenon due to low dissolution ability to SrCO_3_ and tartaric acid. Meanwhile, tartaric acid belonged to H_2_A-type acid. Accordingly, there was secondary ionization equilibrium in the solution.

HOOCCHOHCHOHCOOH↔^−^OOCCHOHCHOHCOOH + H^+^ pKa_1_ = 2.95 (4)

^−^OOCCHOHCHOHCOOH↔^−^OOCCHOHCHOHCOO^−^ + H^+^ pKa_2_ = 3.97 (5)

Obviously, the amount of distilled water affected the pH value of the solution. The ionization degree of tartaric acid was different in solutions with various pH values. On the one hand, the insufficient amount of distilled water restrained the secondary ionization of tartaric acid, which led to difficulty in obtaining clear sol. On the other hand, although additional distilled water helped to dissolve SrCO_3_ and tartaric acid, too much water reduced the collision probability of Ti^4+^ and tartrate ions in solution, which brought about the incomplete complexation reaction.

At the same time, excessive water extended the drying time and increased the cost. Only when the water content was appropriate did the following reactions occur: (a) the SrCO_3_ and tartaric acid fully dissolved, (b) the tartaric acid was completely ionized, and (c) complexation reaction occurred completely. Only when such conditions were met could a clear and transparent homogeneous sol be obtained.

To determine the optimum amount of distilled water (5–25 mL) factors such as the observation of sol transparency, powder agglomeration after sintering, calculation of relative crystallinity, and photocatalytic activity of products were considered.

Table 2 provides the experimental phenomena for SrTiO_3_ and the relative crystallinity determined by adjusting the amount of distilled water. It can be seen from Table 2 that when the amount of distilled water was less than 10 mL, the solubility of SrCO_3_ and tartaric acid was relatively weak, the secondary ionization of tartaric acid was inhibited, and the white precipitate in the sol system could be observed. When the amount of distilled water was 15 mL, the relative crystallinity of the product was still not high despite a transparent sol. When the amount of distilled water was 25 mL, a white precipitate appeared in the sol system, indicating that the distilled water was excessive, that the reaction in the system was incomplete, and that the drying time would be lengthened.

**Figure 2:**
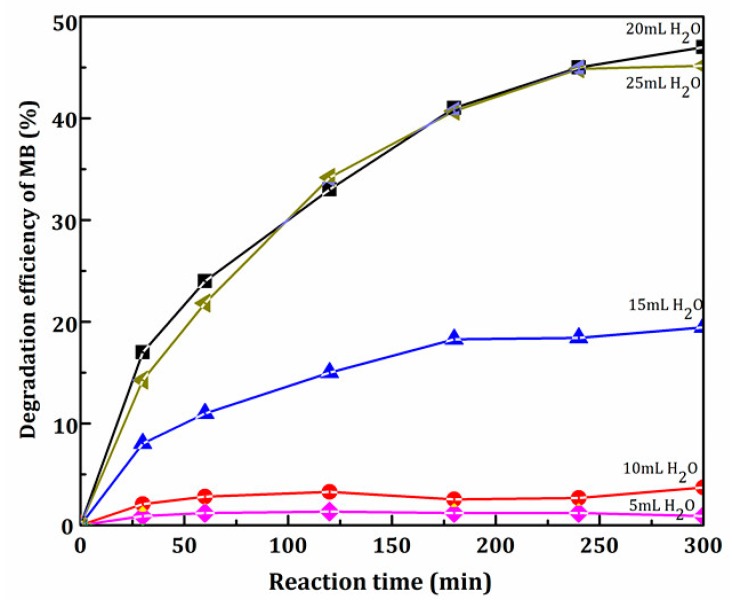
Effect of different dosage of water on MB degradation.

To investigate the influence of the amount of distilled water on the photocatalytic activity of the as-prepared SrTiO_3_, the samples were used for the photocatalytic activity test. Results are shown in Figure 2. It can be seen that when the amount of distilled water was 20 mL, methylene blue had the highest degradation rate. Therefore, the optimum amount of distilled water was found to be 20 mL.

In addition, Table 2 and Figure 2 demonstrate that the higher the crystallinity of the catalyst, the higher the rate of MB degradation.

### 3.2. XRD, IR, and SEM Analysis of SrTiO_3_

**Figure 3:**
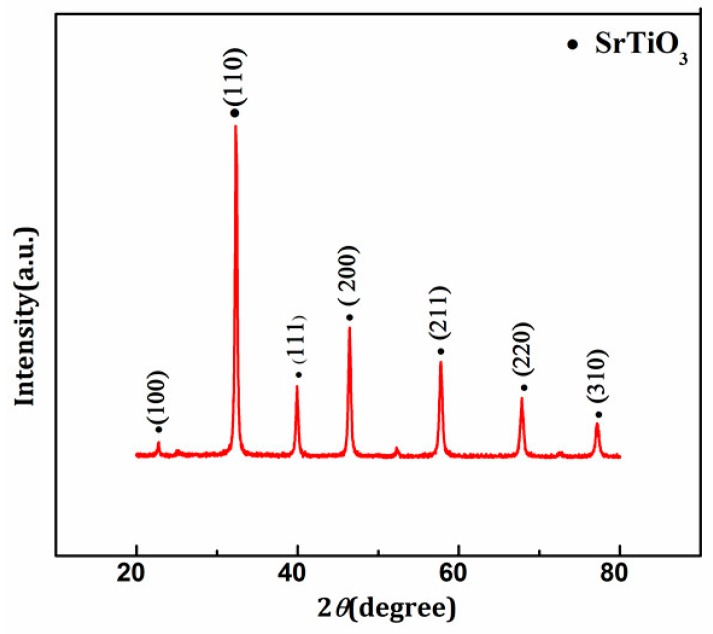
XRD pattern of pure SrTiO_3._

In the preparation process of SrTiO_3_, the molar ratio of Sr^2+^ to tartaric acid was less than 1:4 and the amount of distilled water was 20 mL. The as-prepared SrTiO_3_ was characterized by XRD, IR, BET, and SEM. Figure 3 shows the XRD pattern of SrTiO_3_ prepared under optimum conditions. Compared with the standard card, diffraction peaks at 2θ = 23.0°, 32.4°, 40.4°, 57.8°, 67.8°, 77.2°, and 79.9° belonged to the 100, 110, 111, 200, 211, 220, and 310 of the crystal phase of SrTiO_3_ [10,11,12], respectively. It can be seen that the prepared SrTiO_3_ had a good crystal form and a high diffraction peak intensity. The average particle size of SrTiO_3_ was 20.8 nm calculated by the Debye-Scherrer formula, which reached nanometer level. The crystallinity of SrTiO_3_ was 89%. The BET test results indicated that the specific surface area was 11.8 m^2^/g. The specific surface area was relatively small, which was not conducive to improving photocatalytic activity. Therefore, subsequent experiments to modify SrTiO_3_ to improve its crystallinity and to increase its specific surface area were conducted.

**Figure 4:**
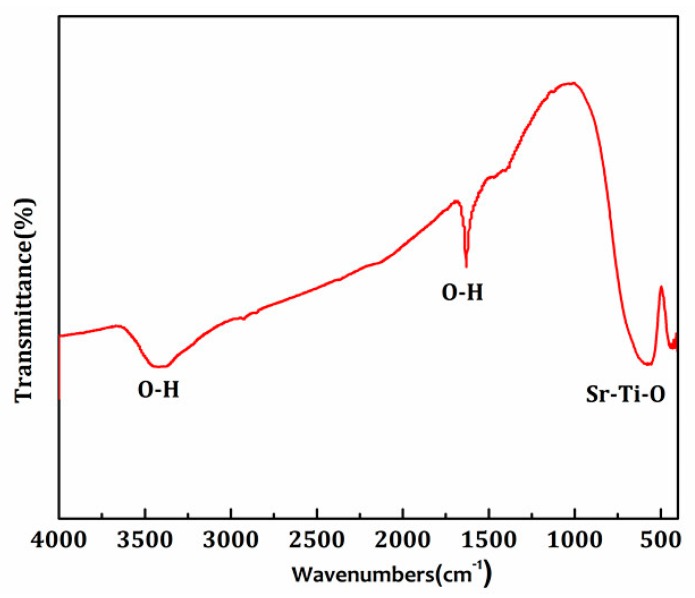
FTIR spectra of pure SrTiO_3_.

Figure 4 displays the FTIR spectrum of SrTiO_3_. The absorption peaks at 3420 cm^−1^ and 1629 cm^−1^ originated from hydroxyl vibration [3]. It is speculated that the hydroxyl group was derived from the adsorption water on the surface of the sample. The absorption peak at 557 cm^−1^ belonged to the characteristic stretching vibration of the Sr-Ti-O bond [11], which confirmed SrTiO_3_ was prepared successfully.

**Figure 5:**
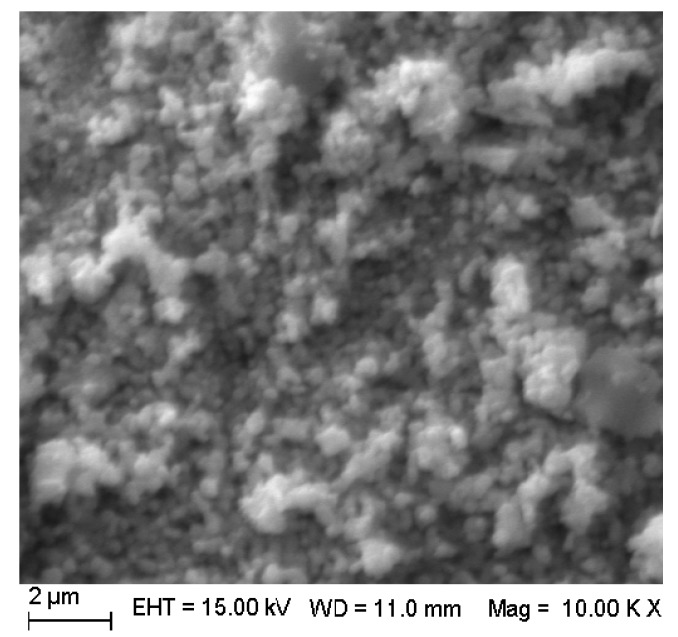
SEM images of pure SrTiO_3_.

It can be seen from Figure 5 that pure SrTiO_3_ particles were spherical. Agglomeration was formed in part as a result of the volatilization or decomposition of organic compounds during the roasting process.

### 3.3. XRD, BET, IR, XPS, and SEM Analysis of Ce^4+^ Doped SrTiO_3_

**Figure 6:**
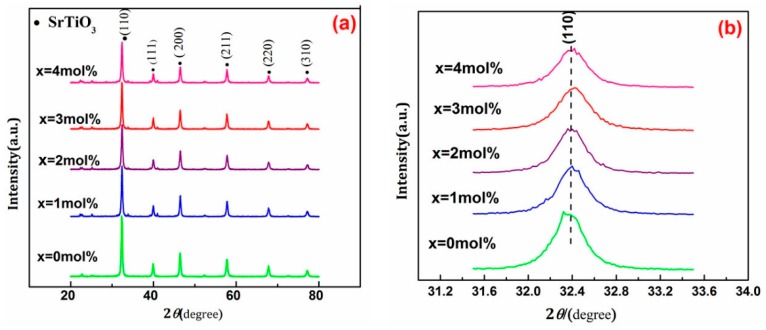
XRD pattern of pure and 1 mol %, 2 mol %, 3 mol %, 4 mol % Ce^4+^ doped SrTiO_3_. (**a**) The whole XRD pattern; (**b**) The local enlargement of the diffraction peaks at 2θ = 32.4°.

From Figure 6a, the diffraction peaks of SrTiO_3_ appear in each of the patterns. The XRD diffraction peaks of Ce^4+^ doped SrTiO_3_ from 1 mol % to 4 mol % were in good agreement with that of pure SrTiO_3_, indicating that the incorporation of Ce^4+^ could not change the crystal structure of SrTiO_3_. There was no Ce^4+^ independent characteristic diffraction peak in the spectra, demonstrating that Ce^4+^ was highly dispersed into SrTiO_3_, even into the crystal lattice of SrTiO_3_. Figure 6b demonstrates that, with the increase of the doping amount of Ce^4+^, the diffraction peak of crystal plane of SrTiO_3_ (110) gradually broadened and shifted to a higher 2θ direction, further indicating that Ce^4+^ entered the framework of SrTiO_3_ [5,6,7] and substituted Sr^2+^ in SrTiO_3_ system. The ionic radius for Ti^4+^, Ce^4+^, and Sr^2+^ showed a higher order of increase from 68 to 113 pm, namely, r_Ti^4+^_ (68 pm) < r_Ce^4+^_ (92 pm) < r_Sr^2+^_ (113 pm). A replacement of Ce^4+^ for Sr^2+^ would reduce the lattice parameter (d) according to the Bragg law (nλ = 2dsinθ, n = 1). The decrease in d values would cause the shift of diffraction peaks to high 2θ direction. The broadening of the manifest diffraction peak indicated that the doped Ce^4+^ ions inhibited grain growth of SrTiO_3_.

The average particle size of samples was calculated by the Debye-Scherrer formula. The average particle size of SrTiO_3_ samples with the doping amount of Ce^4+^ 0~4 mol % were 20.8 nm, 17.2 nm, 13.3 nm, 8.7 nm, and 18.7 nm, respectively. Additionally, compared with pure SrTiO_3_, the specific surface area and pore volume of Ce^4+^ doped SrTiO_3_ samples manifestly increased (See Table 3). The specific surface area of 3 mol % Ce^4+^ doped SrTiO_3_ was more than four times higher than that of pure SrTiO_3_.

Sr^2+^ radius was relatively large, leading to relatively large lattice parameters for a SrTiO_3_ crystal system. Ti^4+^ in the oxygen octahedron system had a good mobility and an inferior stability, which facilitated the fracture of Ti–O bonds. Concurrently, Ti^4+^ produced a positive charge at the grain boundary. The replacement of Sr^2+^ by Ce^4+^ with a smaller radius engendered volume contraction of the unit cell. Thus, the activity scope of Ti^4+^ ion was smaller, and additional Ti–O bonds were not easily broken [13,14], leading to a reduction in the positive charge of the grain boundary. Space contraction was generated, leading to the decrease in grain size and further increasing the specific surface area. However, the grain size of SrTiO_3_ did not continue to decrease with the increase in the content of Ce^4+^. Compared with 3 mol % Ce^4+^ doped SrTiO_3_, the average particle size of 4mol% Ce^4+^ doped SrTiO_3_ slightly increased. When an excess of Ce^4+^ gathered on the surface of SrTiO_3_, a positive charge on the boundary surface increased and space area expanded. Thus, while the crystal grain increased the specific surface area decreased.

**Figure 7:**
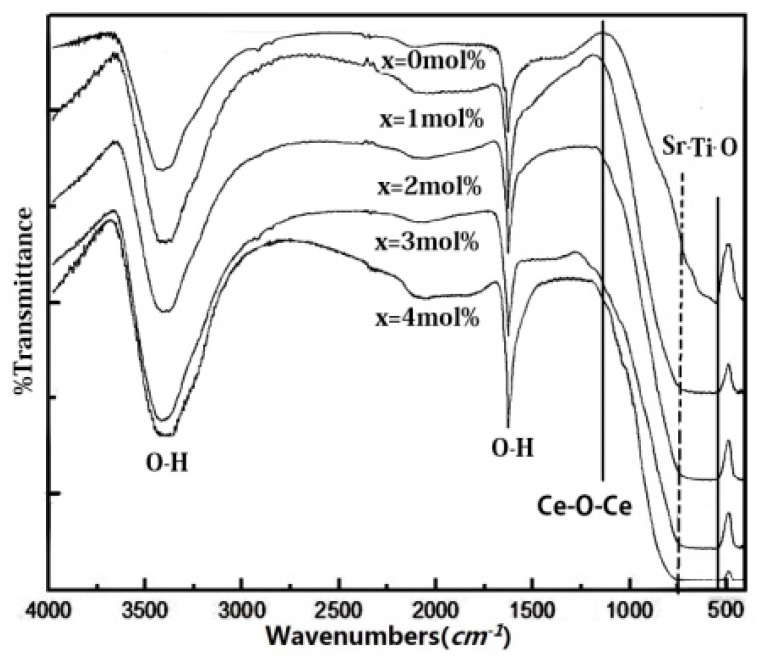
FTIR spectra of pure SrTiO_3_ and 1 mol %, 2 mol %, 3 mol %, 4 mol % Ce^4+^ doped SrTiO_3._

Figure 7 displays the FTIR curves of the samples. FTIR curves of the differing amounts of Ce^4+^ doped SrTiO_3_ all exhibited the characteristic absorption peak of hydroxyl vibration at 3420 cm^−1^ and 1629 cm^−1^ [15]. It is suggested that the hydroxyl group stemmed from the adsorbed water on the surface of the samples. The absorption peaks at 557 cm^−1^ were attributed to the stretching vibration of the Sr–Ti–O bond [14]. No additional peaks appeared at this wavelength range for Ce^4+^ doped SrTiO_3_. The characteristic absorption peak at 1145 cm^−1^ for CeO_2_ was not observed, evincing that doped Ce^4+^ entered into the crystal lattice of SrTiO_3_. According to the XRD test results, the Ti–O bond length shortened after the substitution of Ce^4+^ for Sr^2+^, and the absorption peak of Ti–O bond widened due to the presence of several with a Sr–Ti–O absorption peak.

**Figure 8:**
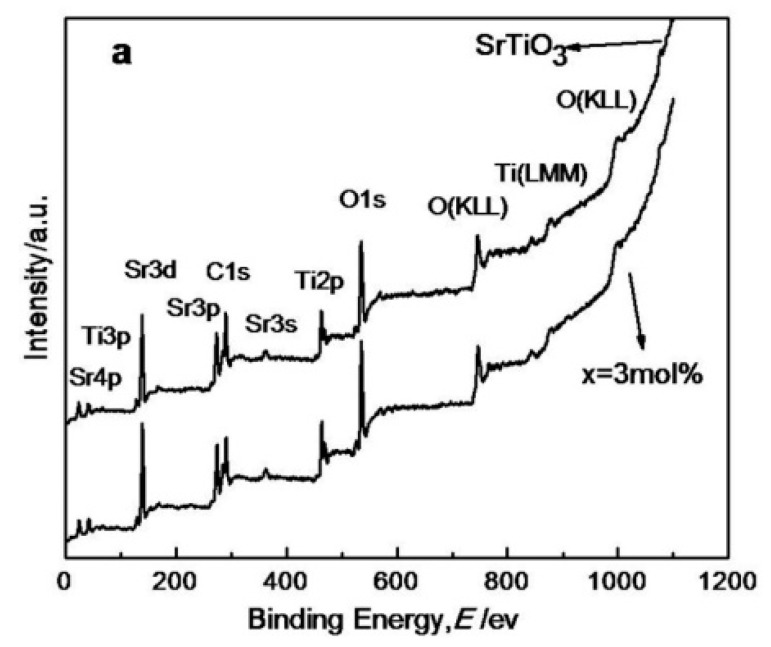

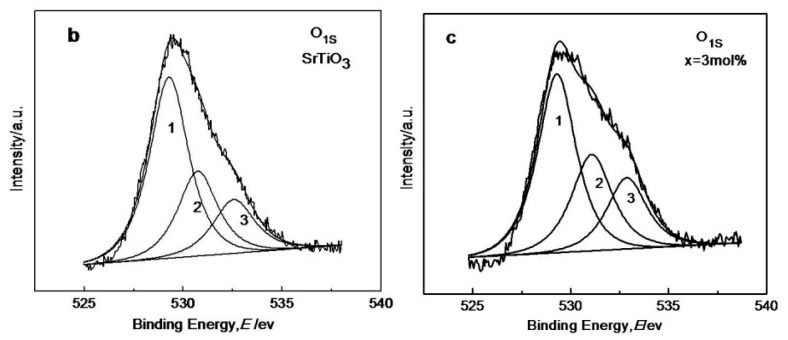
XPS scans for pure and 3 mol % Ce^4+^ doped SrTiO_3_ (**a**) Full scan; (**b**) O_1s_ peaks on pure SrTiO_3_; (**c**) O_1s_ peaks on 3 mol % Ce^4+^ doped SrTiO_3_.

Figure 8 shows the XPS spectra. Figure 8a reveals that the Ce^4+^ was not detected in the 3 mol % Ce^4+^ doped SrTiO_3_, indicating that the Ce^4+^ was indeed highly dispersed in the bulk phase of SrTiO_3_, which is in accordance with the above analysis. Through quantitative analysis (Table 4), n (Sr) /n (Ti) in pristine SrTiO_3_ system was 0.96, possibly signifying the excess Ti^4+^ distributed on the crystal surface. The content of Ti^4+^ decreased from 13.77% to 13.01% after 3 mol % Ce^4+^ doping, also indicating that the reduction of the content of the crystal surface of Ti^4+^ was attributable to the substitution of Ce^4+^ for Sr^2+^. Simultaneously, the O content on the surface of SrTiO_3_ increased. The O_1S_ peak was fitted. The SrTiO_3_ of pure O_1S_ can be divided into three peaks at 529.28, 530.73, and 532.55 eV [8,16,17], respectively, as shown in Figure 8b marked “1”, “2” and “3”. The “1” peak belonged to the characteristic peak of lattice oxygen, and the “2” peak was born of the adsorbed oxygen on the surface. “3” corresponded to the characteristic peak of the physisorption water on the sample surface.

After doping, three peaks for O_1S_ shifted 0.01 eV, 0.35 eV, and 0.31 eV to higher binding energy sites. The smaller atomic radius, the larger both the electronegativity and electron binding energy is. The Ce electronegativity of 1.12 was more than that of Sr (0.95). After the Ce^4+^ replaced Sr^2+^, the electron density around Sr and Ti decreased, the shielding effect was weakened, and the electron binding energy increased. 

A comparison of Figure 8b and 8c demonstrates that the intensity of “2” and “3” peaks increased after doping. The surface oxygen adsorption increased from 26.55% to 28.74% by calculating the area of three peaks. The physical adsorption of H_2_O O_1S_ increased from 17.74% to 20.8%, which was attributed to an increased specific surface area of doped Ce^4+^ as well as enhanced adsorption capacity.

Some adsorbed oxygen were able to capture some free electrons on the surface of SrTiO_3_ to form the active center in the catalytic reaction process. The higher the adsorbed oxygen content on the surface of SrTiO_3_, the higher the catalytic activity was.

**Figure 9:**
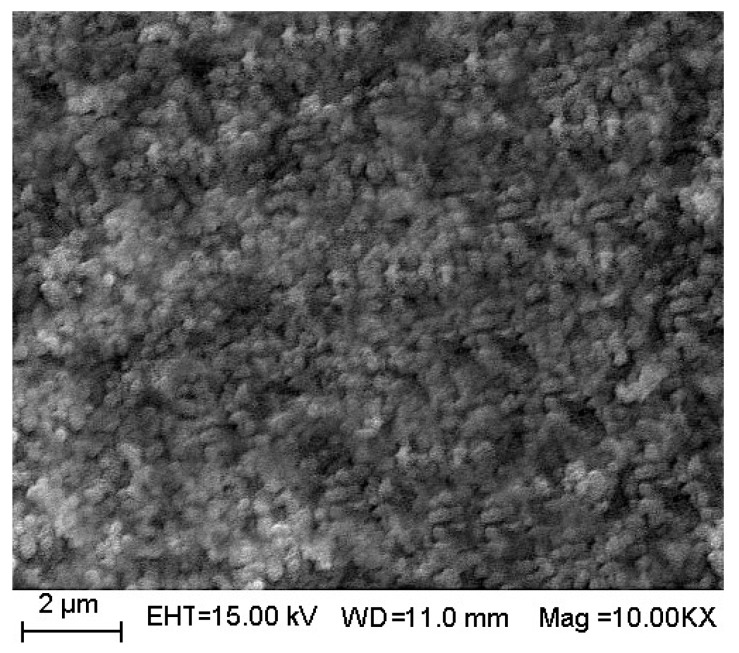
SEM images of 3 mol % Ce^4+^ doped SrTiO_3._

Analysis of the SEM image (See Figure 9) were consistent with those of XRD and BET. It can be seen from Figure 5 that pure SrTiO_3_ particles were spherical. The observed partial agglomeration was due to the volatilization or decomposition of organic material during the roasting process. However, 3 mol % Ce^4+^ doped SrTiO_3_ particles were smaller and had uniform distribution. The agglomeration significantly could be ameliorated.

### 3.4. Optical Properties and Photocatalytic Activity

**Figure 10:**
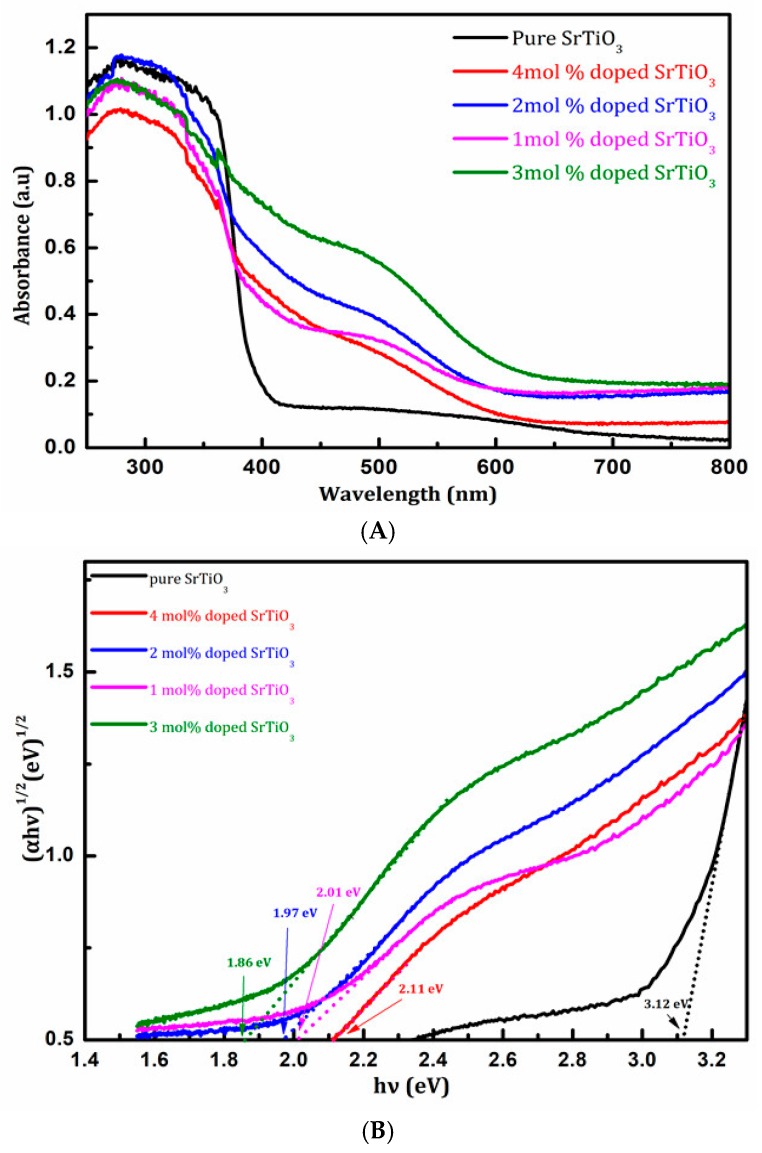
(**A**) UV-vis diffuses reflectance spectra of samples; (**B**) The plot of (Ahv)^2^ vs. hv to estimate the Eg value. (**black**) pure SrTiO_3_; (**purple**) 1 mol % Ce^4+^ doped SrTiO_3_; (**blue**) 2 mol % Ce^4+^ doped SrTiO_3_ (**green**) 3 mol % Ce^4+^ doped SrTiO_3_; (**red**) 4 mol % Ce^4+^ doped SrTiO_3_.

Figure 10 was the UV-Vis diffuse reflectance spectra of pure SrTiO_3_ and Ce^4+^ doped SrTiO_3_ catalysts. It can be seen from Figure 10 that the absorption light region for pure SrTiO_3_ was only in the ultraviolet light range. However, Ce^4+^ doped SrTiO_3_ in 500 nm wavelength produced an observable characteristic absorption peak. The absorption peak could be ascribable to electronic transitions of Ce^4+^ [8]. This further proved that Ce^4+^ had entered into the crystal lattice of SrTiO_3_. In the range of 1–3 mol %, the absorbance intensity gradually increased.

The absorbance of 4 mol % Ce doped SrTiO_3_ in the visible region increased significantly compared with SrTiO_3_. The absorbance of 4 mol% Ce doped SrTiO_3_ in the visible light range was lower than that of 1–3 mol % doping amount. This was attributable to the change of the content of each element and the surface morphology. The results were in conformity with those of XRD BET and SEM tests.

### 3.5. Photocatalytic Activity and Corresponding Mechanism

#### 3.5.1. Effect of Initial Concentration of Methylene Blue on Photocatalytic Activity

**Figure 11:**
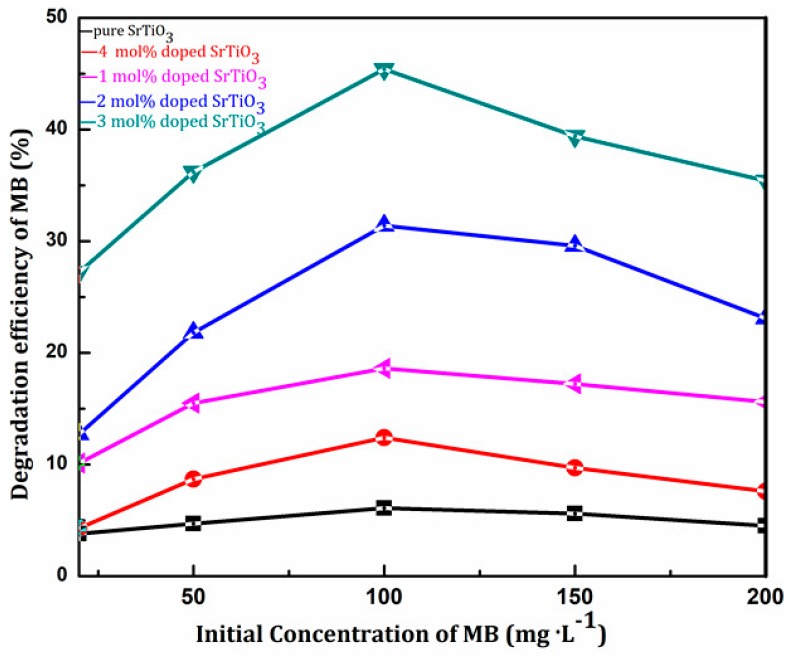
Effect of initial concentration on photocatalytic activity. (**black**) pure SrTiO_3_; (**purple**) 1 mol % Ce^4+^ doped SrTiO_3_; (**blue**) 2 mol % Ce^4+^ doped SrTiO_3_; (**green**) 3 mol % Ce^4+^ doped SrTiO_3_; (**red**) 4 mol % Ce^4+^ doped SrTiO_3_.

It can be seen from Table 5 that the degradation efficiency of 100 mg/L MB after 2 h reaction using 3 mol % Ce doped SrTiO_3_ under visible light irradiation reached 45.4% which was the highest degradation efficiency (See Figure 11).

The lower the initial concentration of methylene blue, the lighter the color was. Thus, the light source had a strong penetrating ability in the solution. However, a lower initial concentration resulted in making concentration diffusion the rate-controlling step in the whole photocatalytic kinetics. In such an occurrence, photocatalytic effect decreased.

However, when the MB concentration was greater than 100 mg/L, the solution color was darker, which significantly obstructed the absorption to visible light and further affected the optimal degradation action. Therefore, the concentration of methylene blue (100 mg/L) was chosen to investigate the photocatalytic activity for the subsequent test.

#### 3.5.2. Photocatalytic Activity and Mechanism

**Figure 12:**
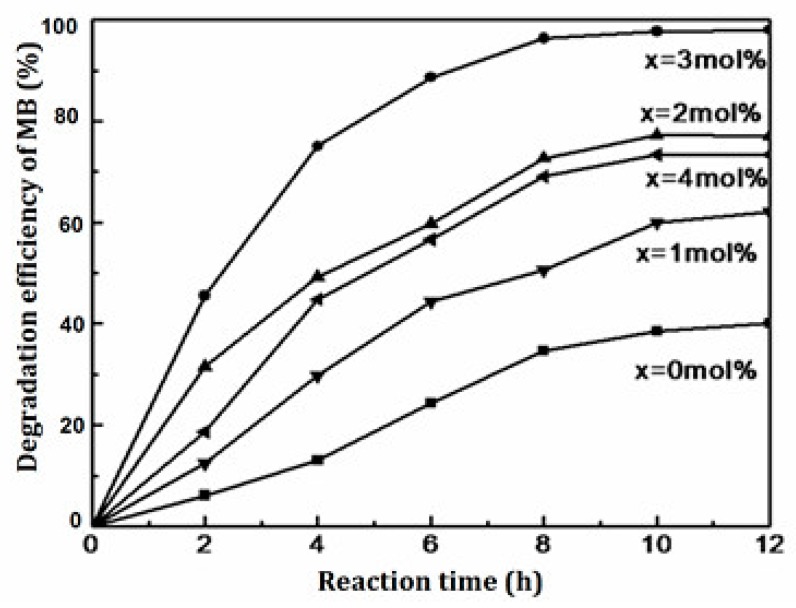
The photocatalytic degradation on MB of 0~4 mol % Ce^4+^ doped SrTiO_3_.

From Figure 12, we can observe the maximum degradation efficiency of methylene blue for pure SrTiO_3_ was only 40%. The photocatalytic activity gradually increased with the increase in doped Ce^4+^ content. The degradation efficiency of methylene blue could reach to 98.7% using 3 mol % Ce^4+^ doped SrTiO_3_.

**Figure 13:**
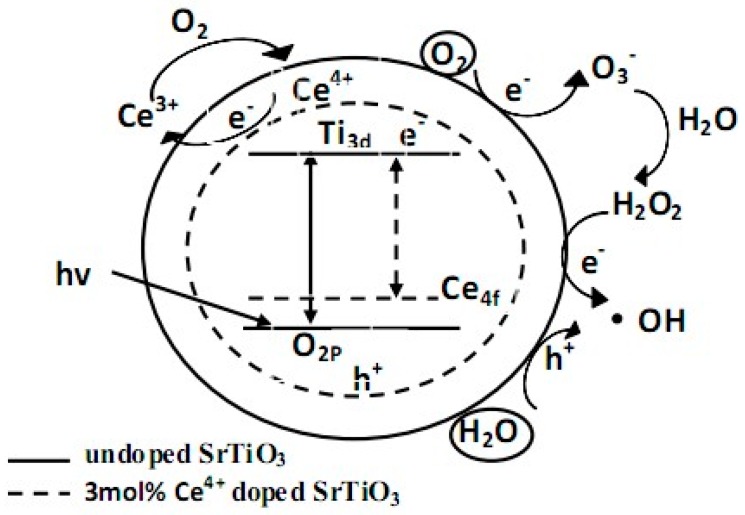
Schematic diagram of photocatalytic mechanism.

(1) According to the photocatalytic mechanism of SrTiO_3_ (Figure 12), under light irradiation, the photogenerated electron and hole separated and moved to different locations on the particle surface to participate redox reactions for organic compounds degradation. The recombination of the photogenerated electron and hole was one of the reasons for the low photocatalytic efficiency of SrTiO_3_. XRD, BET and SEM tests indicated that as the average particle size of Ce^4+^ doped SrTiO_3_ decreased, the specific surface area increased. The photo-excited electron and hole needed to migrate to the surface to play the catalytic role. The smaller the particle size, the shorter migration distance was [18]. Accordingly, the recombination possibility of the photo-excited electron and hole became smaller. In addition, the Ce^4+^ doping improved agglomeration. The surface of the catalyst active center was able to absorb ultraviolet light irradiation [19] which enhanced photocatalytic performance.

(2) Figure 13 shows that the adsorption oxygen on the surface of SrTiO_3_ quickly captured electrons to inhibit recombination of photo-excited electron and hole. Meanwhile, it also could adsorb oxygen and H_2_O to produce ·OH and h^+^. The photo-excited hole could directly transform the surface adsorption of H_2_O into ·OH, which was the active center [20,21]. From the analysis of XPS, Ce^4+^ doping increased the amount of surface adsorption of oxygen and H_2_O. Thus, Ce^4+^ doped SrTiO_3_ could produce more ·OH to enhance photocatalytic ability.

(3) In addition, the UV-vis diffuse reflectance spectrum demonstrated that the band gap width of 3 mol % Ce^4+^ doped SrTiO_3_ became smaller (See Figure 10). This was because the Ce^4+^ doping could introduce the 4f orbital energy level into the midst band gap of SrTiO_3_. On the one side, this reduced the band gap of SrTiO_3_, which made the excitation wavelength of SrTiO_3_ shift from the ultraviolet light range to the visible light range, boosting the utilization rate of visible light. On the other side, this created new energy states, namely, mid gap states, that facilitated the photo-excited electron to jump into the conduction band and to achieve effective separation between the photo-excited electron and the hole. In other words, the doping of Ce^4+^ could create new energy states that could delay the exciton recombination time and could allow charge separation.

(4) Furthermore, the reduction potential of Ce^4+^/Ce^3+^ was 1.61 eV. Ce^4+^ on 4f energy level easily captured electrons to generate Ce^3+^. The generated Ce^3+^ in turn could be oxidized to Ce^4+^ by oxygen on the catalyst surface or in the air [22]. The photoexcited electron was captured continuously by Ce^4+^, which further inhibited the recombination between the photo-excited electron and the hole.

In comparison to 3 mol % Ce^4+^ doped SrTiO_3_, photocatalytic activity of 4 mol % Ce^4+^ doped SrTiO_3_ decreased. An excessively doped Ce^4+^ increased the size of the catalyst particle size. Moreover, n (Sr)/n (Ti) in the Ce^4+^ doped SrTiO_3_ increased (See Table 4), indicating that excessive doping brought out the generation of SrO on the surface of SrTiO_3_, which impeded the light absorption for SrTiO_3_ and affected its catalytic activity.

## 4. Conclusions

(1) The tartaric acid aqueous solution is used as a solvent, dispersant, and stabilizer to prepare SrTiO_3_ using the sol–gel method. The improved preparation method has some advantages, for example, the simplicity of the process with less equipment, its short preparation time, and its low cost. When 1.47 g SrCO_3_ was weighted to synthesize SrTiO_3_, the optimum molar ratio of Sr^2+^ and tartaric acid was 1:4. The optimum amount of distilled water was 20 mL. In practical application, we can adjust the molar ratio (Sr^2+^—tartaric acid) and the amount of water on the basis of the quality of SrCO_3_.

(2) The degradation efficiency of methylene blue reached 98.7% using 3 mol % Ce^4+^ doped SrTiO_3_, indicating it had an excellent photocatalytic activity. The introduction of Ce^4+^ into the SrTiO_3_ revealed several prominent advantages: (i) The Ce^4+^ doping increased specific surface area more than four times compared with pure SrTiO_3_; (ii) The doped Ce^4+^ enhanced the physical adsorption of H_2_O and adsorbed oxygen on the surface of SrTiO_3_ to produce more catalytic active centers; (iii) The incorporation of Ce^4+^ into the crystal lattice for SrTiO_3_ created mdi gap states that delay the exciton recombination time and allow for charge separation; (iv) Ce^4+^ repeatedly captured photoelectron to produce Ce^3+^, which inhibited the recombination of photogenerated electrons and holes.

## Figures and Tables

**Table 1:** Experimental phenomena and relative crystallinity of SrTiO_3_ in the system with different molar ratios of Sr^2+^ and tartaric acid.

Sample Number	n_(Sr^2+^)_/n_(tartaric acid)_	Sol State	Relative Crystallinity (%)
1	2:1	white precipitation	23
2	1:1	white precipitation	27
3	1:2	transparent	78
4	1:3	transparent	85
5	1:4	transparent	89
6	1:5	transparent	89

**Table 2:** Experimental phenomena and relative crystallization of SrTiO_3_ prepared with different amounts of water.

Sample Number	H_2_O (mL)	Sol State	Drying Time (h)	Relative Crystallinity (%)
1	5	white precipitation	5	13
2	10	white precipitation	6	20
3	15	transparent	8	80
4	20	transparent	10	89
5	25	white precipitation	15	89

**Table 3:** Specific surface area, pore volume, and average pore diameter of the samples.

Catalysts	Surface Area/(m^2^·g^−1^)	Pore Volume/(cm^3^·g^−1^)	Mean Pore Size/nm
SrTiO_3_	11.8	0.040	13.6
1 mol % Ce^4+^ doped SrTiO_3_	13.5	0.047	14.2
2 mol % Ce^4+^ doped SrTiO_3_	25.9	0.052	8.1
3 mol % Ce^4+^ doped SrTiO_3_	48.7	0.132	7.7
4 mol % Ce^4+^ doped SrTiO_3_	12.7	0.048	15.2

**Table 4:** Surface atomic composition and atomic ratio of catalysts.

Catalysts	Surface Atomic Composition			Surface Atomic Ratio
	Sr	Ti	O	n(Sr)/n(Ti)
SrTiO_3_	24.18%	13.77%	55.44%	0.96
3 mol %Ce^4+^ doped SrTiO_3_	25.02%	13.01%	57.19%	1.05

**Table 5:** Effect of initial concentration on photocatalytic activity.

Samples	Degradation Efficiency (%)				
	20 mg/L	50 mg/L	100 mg/L	150 mg/L	200 mg/L
SrTiO_3_	3.8	4.7	6.1	5.6	4.5
1 mol % Ce^4+^ doped SrTiO_3_	10.1	15.5	18.6	17.2	15.6
2 mol % Ce^4+^ doped SrTiO_3_	12.7	21.8	31.4	29.6	23.1
3 mol % Ce^4+^ doped SrTiO_3_	27.2	36.2	45.4	39.4	35.4
4 mol % Ce^4+^ doped SrTiO_3_	4.3	8.7	12.4	9.7	7.6
